# Uropathogens and their antibiotic susceptibility patterns among diabetic patients at st. john of god hospital, duayaw nkwanta, Ghana: a cross‐sectional study

**DOI:** 10.1002/hsr2.70072

**Published:** 2024-09-18

**Authors:** Abdul‐Karim Iddrisu, George Owusu, Samuel Kofi Doe, Augustine Apraku Yeboah, Joseph Agyapong, Nicholas Yankey

**Affiliations:** ^1^ Department of Medical Laboratory Science University of Energy and Natural Resources Sunyani Ghana; ^2^ Department of Mathematics and Statistics University of Energy and Natural Resources Sunyani Ghana

**Keywords:** antibiotics, diabetic patients, Duayaw Nkwanta, susceptibility, uropathogen

## Abstract

**Background:**

Uropathogens are microorganisms that cause urinary tract infections (UTIs). Owing to higher blood glucose levels and compromised immune functions, treatment of uropathogens in diabetic patients is a challenge.

**Aim:**

This study aims to assess the prevalence of uropathogens and their antibiotic susceptibility among diabetic patients at St. John of God Hospital at Duayaw Nkwanta (SJGHDN) in the Ahafo region of Ghana.

**Methods:**

The cross‐sectional study recruited 175 diabetic patients at SJGHDN between August and September 2023. Questionnaires were used to collect patients’ background information. Fasting Blood Sugar (FBS) was assessed by using a glucometer. Urine samples were examined for the presence of uropathogens. A sterile inoculating loop with a calibrated volume of 2 µl was used for plating. Each colony equals 500 CFU/mL. Significant uropathogen was determined by multiplying the counted colonies by 500 to obtain CFU/mL. Positive uropathogen was defined as CFU ≥ 10^5^/mL. Significant uropathogen was defined as ≥200 colonies per sample. The disc diffusion method was used to determine antibiotic susceptibility.

**Results:**

Out of the 175 patients, 19.4% expressed various uropathogens with *Escherichia coli* being the predominant. Suboptimal glucose level was the most significant risk factor (*p* = 0.038). Glucosuria (*p* = 0.036), hazy urine (*p* = 0.028), positive leukocyte esterase (*p* = 0.001), and pus cells in urine sediment (*p* = 0.020) were significant indicators of uropathogen occurrence. *Klebsiella pneumonia* and *Proteus mirabilis* were resistant to ≥4 antibiotics. Amikacin, nitrofurantoin, levofloxacin, ciprofloxacin, and ceftriaxone demonstrated efficacy against the isolates.

**Conclusion:**

This study underscores the notable prevalence of uropathogens in diabetic patients and the alarming levels of antibiotic resistance observed. The results highlight the critical need for vigilant monitoring and customized treatment approaches, particularly for diabetic patients exhibiting risk factors such as elevated urine glucose levels, cloudy urine, and presence of leukocyte esterase and pus cells in urine sediment. The significant resistance to frequently used antibiotics like co‐trimoxazole and tetracycline points to the necessity of routine susceptibility testing and the use of alternative antibiotics for effective treatment. These findings can assist healthcare providers in more effectively managing and preventing UTIs in diabetic populations.

## 1 INTRODUCTION

Diabetes mellitus (DM) is a condition often characterized by chronic hyperglycemia. The rise in sugar levels in the blood provides a favorable environment for the growth and multiplication of uropathogens.^1^ Consequently, UTIs have become one of the common complications of DM and the development of resistance to antibiotics due to impaired immune functions in diabetic patients has become a global health challenge.^2^ Several studies have established a clear association between type 2 DM (T2DM) and recurrent UTIs (rUTIs). For instance, a systematic review and meta‐analysis by Carrillo‐Larco et al.^3^ confirmed that T2DM was the most significant risk factor for rUTIs. Their study recruited 61 articles from 21 countries and involved 449 247 T2DM patients. Papp and colleagues^4^ also conducted a systematic review of 15 articles from 10 countries and reported that hyperglycaemia was the number one risk factor for rUTIs, and the rate of occurrence could be as high as 23.4% to 37% in women. Coincidentally, the studies identified *Escherichia coli (E. coli), Enterococcus species (Enterococcus. spp), Klebsiella pneumoniae (K. pneumoniae), Proteus mirabilis (P. mirabilis), Pseudomonas aeruginosa (P. aeruginosa), Staphylococcus aureus (S. aureus), Coagulase‐negative staphylococci (CoNS), and Enterobacter species (Enterobacter. spp)* as the common uropathogens involved in the rUTIs in T2DM individuals.^3^, ^4^, ^5^, ^6^, ^7^ Though diabetes, uropathogens, and rUTIs are global health threats, low‐income countries (LICs) are affected most due to socio‐cultural and socioeconomic diversities, as well as poor diagnosis and treatment as a result of resource limitations.^8^, ^9^ However, the magnitude of occurrence of uropathogens and rUTIs among T2DM patients in the LICs is underexplored.^10^ In Ghana, the prevalences of T2DM and uropathogens in T2DM patients are reported to be 3.95% and 9.2% respectively.^1^, ^11^ Due to cultural and economic diversities, managing this condition is more challenging. For instance, some patients in remote areas do not go for regular check‐ups and treatments because they cannot afford transportation expenses. Also, poor hygienic practices such as drinking contaminated water, eating contaminated food, and sharing public toilet facilities that are sometimes not sanitized, could enhance the spread of resistant uropathogens. Though the intersection of T2DM and the occurrence of uropathogens and rUTIs in Ghana has been reported, there is still a knowledge gap on the contribution of the health facilities, hygienic practices, and health‐seeking behavior of T2DM patients to the occurrence of uropathogens and rUTIs. Also, there is limited data regarding the reliable predictors of the occurrence of uropathogens and rUTIs in patients. Given the above, this study aims to explore the prevalence of UTI and the antimicrobial resistance patterns among diabetic patients in Ghana. It will also examine whether health‐seeking behaviour and sanitation standards affect the study cohort's spread of uropathogens and rUTIs. The novelty of this research lies in its comprehensive approach to unraveling the relationship between uropathogens, rUTIs, and sanitation standards among T2DM patients in the study setting, which has been underexplored in the existing literature. The findings of this study have the potential to inform targeted interventions, guide policy formulation, and ultimately improve the quality of life for diabetic patients in Ghana. Through this research, we aim to contribute to the global discourse on T2DM and UTIs, providing valuable insights that can be applied to similar contexts worldwide.

## 2 MATERIAL AND METHODS

### 2.1 Study design

A cross‐sectional study was conducted between August and September 2023 on diabetic patients receiving treatment at SJGHDN in the Ahafo region of Ghana. Participants were confirmed diabetes type 2 clients at the Hospital. Diagnoses were made based on fasting glucose levels of 126 mg/dL or higher and HbA1c levels of 6.5% or higher.^12^, ^13^ These patients have unique ID cards. A convenient sampling technique was used to recruit study participants.

### 2.2 Inclusion criteria

The study exclusively enrolled individuals ≥18 years old with confirmed type 2 DMs patients attending the diabetic review clinic between August and September 2023.

### 2.3 Sample size calculation

The minimum sample size for the study was determined by using the Cochran formula *n* = z^2^* p (1‐p)/m^2^, where *n* = minimum sample size, z = standard normal variance = 1.96 to obtain a power of 95% confidence interval and a type 1 error probability of 5%. *P* = average prevalence of UTI was 9.2% according to Forson et al. (2021).^11^

$$n=\frac{1.96^{2}\times 0.092(1-0.092)}{0.05^{2}}$$

n=(3.8416* 0.083536)/0.0025

n = 128.36

For the study, a minimum sample size of 129 diabetics was significant. A total of 175 diabetics meeting the inclusion criteria were enrolled to increase the study's statistical power.

### 2.4 Exclusion criteria

Individuals with DM who were presently undergoing antibiotic treatment for any medical condition were excluded from this study to prevent potential confounding factors associated with ongoing antibiotic therapy.

### 2.5 Socio‐demographic data collection and fasting blood sugar determination

Informed consent was obtained from each participant. Socio‐demographic data was collected using structured questionnaires. The data obtained included duration of DM, comorbidities, type of toilet facility used, presence or absence of symptoms of UTI, and usage of antibiotics. Thereafter, FBSlevels were measured using a glucometer (YASEE® 719, USA). The study protocol conformed to the ethical guidelines of the 1975 Declaration of Helsinki as reflected in a prior approval by the institution & # 39; human research committee.

### 2.6 Urine sample collection and laboratory tests

#### 2.6.1 Urinalysis

Participants were given two sterile urine containers labeled A and B to produce clean, midstream urine. For the specimen in container A, the semi‐quantitative assessment of urine leucocytes, glucose, protein, nitrites, pH, and specific gravity was conducted using Mission® Combi‐Ten urine biochemistry strips. Subsequently, the urine sample was centrifuged at 1500 revolutions per minute (rpm) for 5 min.^14^ The supernatant was meticulously discarded, and a single droplet of the sediment was delicately placed onto a standard microscope slide and covered with a 22 × 22 mm coverslip. The examination of the urine specimens for the presence of pus cells, red blood cells, epithelial cells, and *Trichomonas vaginalis* was performed using the low objective lens (x40) of the Olympus® CX 21 microscope and recorded.

#### 2.6.2 Urine culture

Cysteine lactose electrolyte‐deficient (CLED) agar was used as a culture medium based on its ability to demonstrate morphologic differentiation of colonies and prevent *Proteus species*. from swarming. As a nutrient agar, it supports the growth of all uropathogens and has demonstrated 100% agreement with other standard agars used in isolating uropathogens. CLED has widely been used in other works of similar nature.^15^, ^16^ Briefly, urine specimens in container B were plated on the CLED agar plate. A sterile inoculating loop with a calibrated volume of 1/500 ml (2 µl) was used.^14^ A loop full of urine specimens was streaked on a CLED agar plate using the quarter plating technique. Urine specimens with a cloudy appearance were limited to three specimens per agar plate. The plate was incubated at 37°C for 24 h. After the incubation, agar plates were observed for growth, and colonies were observed for their lactose fermentation pattern and colony morphology. The colonies were counted, and significant uropathogen was determined by multiplying the counted colonies by 500 to obtain colony‐forming units per ml (CFUs/ml). The threshold for the definition of a significant uropathogen is 10^5^ CFU/mL, which is approximately equivalent to 100,000 CFU/ml. Positive uropathogen was defined as CFUs ≥10^5^/ml. Colonies that showed significant bacteriuria were identified using colony morphology, gram stain reaction, and biochemical tests such as the Triple Sugar Iron test, citrate test, oxidase test, indole test, catalase test, coagulase test, and urease test.

#### 2.6.3 Disk diffusion test

Mueller Hinton Agar was the agar of choice for all susceptibility tests performed in this study. The Kirby Bauer disk diffusion method was employed based on the Clinical and Laboratory Standards Institute (CLSI) guidelines for the conduct of antibiotic susceptibility test.^17^ From a 24‐h pure culture, 4–6 bacterial colonies were gathered using a sterile loop and transferred to a tube containing 2 ml of sterile normal saline. The mixture was gently agitated to achieve a uniform suspension, whose turbidity matched a 0.5% McFarland standard. A sterile cotton swab was dipped into the saline suspension and pressed against the side of the tube before being used to streak and cover the entire surface of the plate. Axiom® multidiscs for urinary isolates were evenly placed on the inoculated agar plates. The antibiotics used in the susceptibility tests were: ampicillin/sulbactam (20 µg), cotrimoxazole (25 µg), amikacin (30 µg), chloramphenicol (30 µg), cephalexin (30 µg), tetracycline (30 µg), ciprofloxacin (5 µg), nitrofurantion (300 µg), ceftriaxone (30 µg), levofloxacin (5 µg), norfloxacin (10 µg), and ofloxacin (5 µg). The plates were incubated in air at 37°C for 24 h. The diameter of the zone of inhibition was measured after 24 h of incubation. The zone of inhibition was classified into sensitive, intermediate sensitive, and resistant.

### 2.7 Data analysis

Data was entered into Microsoft Excel and analyzed using the Statistical Package for Social Sciences version 26.0 (Chicago, United States of America). For categorical data, numbers and proportions for each category were presented and the mean was used to represent the continuous age of participants. The chi‐square test statistic^18^, ^19^, ^20^ was used to assess the significant association between uropathogens presence and patients’ characteristics. We identified risk factors of uropathogens presence using a multivariate logistic regression model.^21^, ^22^, ^23^ Statistical significance was defined as *p* < 0.05.

## 3 RESULTS

### 3.1 Socio‐demographic characteristics of study participants

Table 1 presents the percentage distribution of the socio‐demographic characteristics of the study participants. Out of the 175 participants, 144 (82.3%) were females and 31 (17.7%) were males. The mean age of the participants was 60.89 ± 12.81 years. The majority of diabetes patients, 96 out of 175 (54.9%), are between the ages of 50 and 69.

**Table 1:** Socio‐demographic variables of participants.

VARIABLE	Number (N)	Mean ∓SD	Percentage (%)
Age Group (Years)			
30–49	33		18.86
50–69	96		54.86
70 and above	46		26.29
Gender			
Male	31		17.71
Female	144		82.29
Age		60.89∓12.81	

*SD: Standard deviation.

### 3.2 Lifestyle and clinical features of the study participants

The results in Table 2 showed that 126 (72%) and 128 (73.1%) of the study participants respectively used private toilet facilities and water closets (WC). Per the FBS results, 76 (43.4%) of them had suboptimal glycemic control, 61 (34.9%) had poor glycemic control and 38 (21.7%) had optimal glycemic control with FBS 3.5–6.5 mmol/l. One hundred and thirty‐six (136) (76.7%) had one or more co‐morbidities, and 39 (22.3%) did not have any co‐morbidity. The predominant co‐morbidity was hypertension, which was present in 130 (74.3%) participants. Out of 175 participants, 125 (71.4%) were asymptomatic and 50 (28.57%) of them exhibited symptoms such as frequent urination, painful urination, pruritus, and oliguria. Urine glucose levels of +4, trace, +3, +2, and +1 were respectively observed among 23 (13.1%), 11(6.3%), 9 (5.1%), 7 (4%), and 4 (2.3%) participants.

**Table 2:** Lifestyle and Clinical Factors.

VARIABLES	FREQUENCY (*n* = 175)	PERCENTAGE (%)
Type of Toilet Facility		
Public	49	28.00
Private	126	72.00
Toilet Facility		
KVIP	47	26.86
WC	128	73.14
FBS (mmol/l)		
Optimal (3.5–6.5)	38	21.71
Suboptimal (6.6–10)	76	43.43
Poor (Above 10)	61	34.86
Co‐Morbidity		
No	39	22.29
Yes	136	77.71
Nature of Co‐Morbidity		
Hypertension	130	74.29
Prostatitis	1	0.57
Hypertension and Ulcer	3	171
Hypertension and Fever	1	0.57
Hypertension and Arthritis	1	0.57
Symptoms of UTI		
None	125	71.43
Frequent urination	28	16.00
Painful urination	10	5.71
Pruritus	4	2.29
Oliguria	8	4.57
Urine Glucose (mg/dL)		
Negative	121	69.14
+1	7	4.00
+2	4	2.29
+3	9	5.14
+4	23	13.14
Trace	11	6.29

This presents the lifestyle and clinical features of interest among the study population.

Abbreviations: KVIP, Kumasi ventilated improved pit (public toilet); WC, water closet.

### 3.3 Prevalence of uropathogens

Table 3 presents the results of uropathogen prevalence. Out of the total of 175 study participants, 34 (19.4%) tested positive for uropathogens. The most prevalent uropathogens isolated were *E. coli;* 17 (50%), followed by *Enterococcus spp;* 5 (14.7%), *and CoNs;* 4 (11.8%). A total of 34 culture‐positive isolates belonging to Gram‐negative Rods and Gram‐positive Cocci were identified.

**Table 3:** Summary of Uropathogens Isolate.

Isolates	Frequency (*n* = 34)	Percentage (%)
**Gram Negative Rods**		
*Escherichia coli*	17	50.00
*Klebsiella pneumoniae*	1	2.94
*Proteus mirabilis*	1	2.94
*Pseudomonas aeruginosa*	2	5.88
*Enterobacter spp.*	2	5.88
**Gram Positive Cocci**		
*Enterococcus spp.*	5	14.71
*Staphylococcus aureus*	2	5.88
*Coagulase negative staphylococci*	4	11.76
**Total**	**34**	**100**

### 3.4 Socio‐demographic, lifestyle and clinical predictors of the presence of uropathogens

The unadjusted odds ratio (unaOR), adjusted odds ratio (aOR), p‐value and 95% confidence interval (95% CI) from the multivariate logistic regression model are presented in Table 4. These results showed reduced risk (aOR = 0.122, 95% CI: 0.017–0.887, *p*‐value = 0.038) of occurrence of uropathogen among patients with suboptimal FBS levels (6.6–10 mmol/L) compared with optimal FBS level (3.5–6.5 mmol/L). We observed 11.2‐fold increased risk (aOR = 11.212, 95% CI: 1.168–107.594, *p* = 0.036) of uropathogen occurrence among participants whose urine glucose was +3 compared with those who had negative urine glucose. Participants with hazy urine appearance had a 14.9‐fold increased risk (aOR = 14.896, 95% CI: 1.347–164.728, *p*‐value = 0.028) of uropathogens occurrences compared with those with clear urine appearance. There was 18.5‐fold increased risk (aOR = 18.479, 95% CI: 2.344–145.670, *p*‐value = 0.006) of uropathogen occurrence among study participants with +3 leukocyte esterase compared with those who have negative leukocyte esterase and 25.9‐fold increased risk (aOR = 25.915, 95% CI: 3.508–191.454, *p*‐value = 0.001) of uropathogen occurrence among study participants with leukocyte esterase trace compared with those who have negative leukocyte esterase.

**Table 4:** Socio‐demographic, lifestyle, and clinical predictors of the presence of uropathogens.

VARIABLES	unaOR (95% CI)	‐value	aOR (95% CI)	p‐value
Gender				
Male	1	‐	1	‐
Female	3.715(1.070–12.896)	0.039	5.85(0.511–66.887)	0.156
Age (year)				
30–49	1	‐	1	‐
50–69	1.585(0.586–4.286)	0.365	0.729(0.133–3.994)	0.716
70 and above	1.773(0.594–5.288)	0.305	0.953(0.141–6.434)	0.962
Toilet Facility				
KVIP	1.600(0.764–3.354)	0.21	0.455(0.095–2.184)	0.325
WC	1	‐	1	‐
Fasting Blood Sugar (mmol/L)				
Optimal (3.5–6.5)	1	‐	1	‐
Suboptimal (6.6–10)	0.578(0.226–1.475)	0.251	0.122(0.017–0.887)	**0.038** *
Poor (Above 10)	1.470(0.601–3.596)	0.399	0.739(0.098–5.562)	0.769
Urine Glucose (mg/dL)				
Negative	1	‐	1	‐
+1	6(1.253–28.742)	0.025	6.111(0.174–214.973)	0.319
+2	1.500(0.149–15.109)	0.71	1.191(0.022–64.186)	0.931
+3	3.600(0.893–14.505)	0.072	11.212(1.168–107.594)	**0.036** *
+4	1.588(0.562–4.489)	0.383	3.652(0.521–25.589)	0.192
Trace	7.875(2.120–29.258)	0.002	12.521(0.940–166.836)	0.056
Urine Appearance				
Clear	1	‐	1	‐
Hazy	14.333(2.914–70.493)	0.001	14.896(1.347–164.728)	**0.028** *
Leukocyte				
Negative	1	‐	1	‐
+1	2.374(0.445–12.653)	0.311	0.566(0.027–11.679)	0.713
+2	16.615(4.915–56.167)	<0.001	18.479(2.344–145.670)	**0.006** **
+3	49.846(5.557–447.083)	<0.001	1.587(0.016–157.572)	0.844
Trace	10.800(3.952–29.515)	<0.001	25.915(3.508–191.454)	**0.001** **
Pus Cells				
0	1	‐	1	‐
1	4.519(1.283–15.915)	0.019	1.421(0.235–8.597)	0.702
2	4.700(1.199–18.420)	0.026	6.417(0.852–48.316)	0.071
3	17.625(4.998–62.148)	<0.001	9.785(1.663–57.570)	**0.012** *
4	11.750(0.670–206.048)	0.092	17.762(0.183–1728.345)	0.218
5	29.375(4.896–176.234)	<0.001	46.188(1.817–1174.219)	**0.020** *

The table shows how socio‐demographic, lifestyle, and clinical variables could be used to predict the presence of uropathogens. Suboptimal glucose levels (6.6–10 mmol/L), urine glucose of +3, hazy urine, trace and +2 levels of urine leukocytes, and pus cells of +3 and +5 were found to be significant predictors of the presence of uropathogens.

* *p*‐value < 0.05;

** *p* value < 0.01.

Study participants with pus cells of 3/HPF are associated with 9.8‐fold increased risk (aOR = 0.785, 95% CI: 1.663–57.570, *p*‐value = 0.012) of uropathogen occurrence relative to those with no pus cells. The results also revealed a 46‐fold increased risk (aOR = 46.188, 95% CI: 1.817–1174.219, *p*‐value = 0.020) of uropathogen occurrence among those with pus cells of 5/HPF compared with those who have no pus cells.

### 3.5 Drug susceptibility pattern of uropathogens

**Figure 1:**
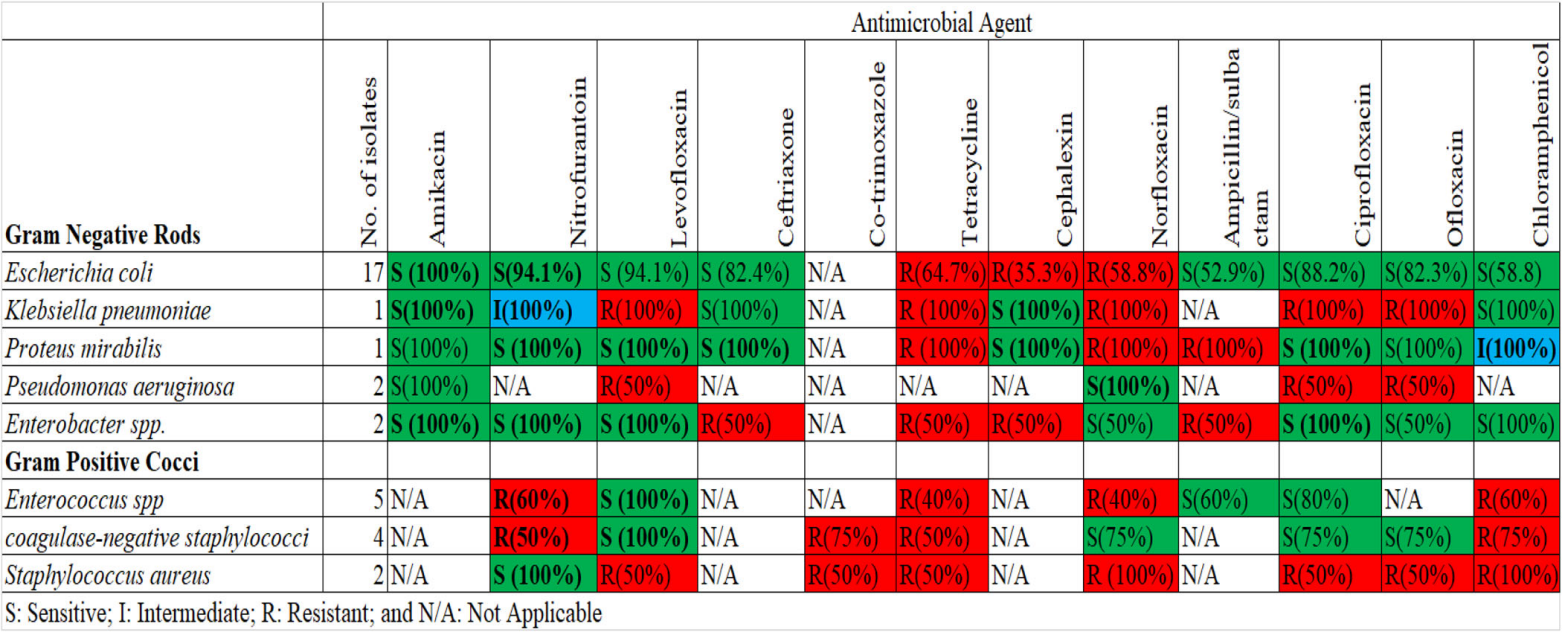
Antibiogram susceptibility pattern for Gram‐negative and Gram‐positive bacteria. *K. pneumoniae* was 100% resistant to levofloxacin, tetracycline, norfloxacin, ciprofloxacin, and ofloxacin. *P. mirabilis* was 100% resistant to tetracycline, norfloxacin, and ampicillin/sulbactam. *S. aureus* was 100% resistant to norfloxacin and chloramphenicol.

The susceptibility patterns of uropathogens are illustrated in Figure 1. The overall percentages of drug‐resistant uropathogens were as follows: *E. coli* at 18.18% (2 out of 11), *P. aeruginosa* at 60% (3 out of 5), *Enterobacter spp*. at 36.36% (4 out of 11), *K. pneumoniae* at 50.00% (5 out of 10), *P. mirabilis* at 27.27% (3 out of 11), *Enterococcus spp*. at 57.14% (4 out of 7), *CoNS* at 50.00% (4 out of 8), and *S. aureus* at 87.50% (7 out of 8).

The Gram‐negative isolates showed 100% susceptibility to amikacin. Specifically, *E. coli* exhibited high susceptibility to amikacin (100%), nitrofurantoin (94.1%), levofloxacin (94.1%), ceftriaxone (82.4%), ciprofloxacin (88.2%), and ofloxacin (82.3%), but showed resistance to tetracycline (64.7%) and cephalexin (35.3%). *E. coli* was susceptible to 81.81% (9 out of 11) of the antibacterial agents. *P. mirabilis* was susceptible to 63.63% (7 out of 11) of the antibacterial agents, showing 100% susceptibility to amikacin, nitrofurantoin, levofloxacin, ceftriaxone, cephalexin, ciprofloxacin, and ofloxacin. *Enterobacter spp*. was susceptible to 63.63% (7 out of 11) of the antibacterial agents, with 100% susceptibility to amikacin, nitrofurantoin, levofloxacin, ciprofloxacin, and chloramphenicol. *K. pneumoniae* was 100% susceptible to amikacin, ceftriaxone, cephalexin, and chloramphenicol. *P. aeruginosa* showed 100% susceptibility to amikacin and norfloxacin.

Among the Gram‐positive isolates, Enterococcus spp. was 100% susceptible to levofloxacin, 80% to ciprofloxacin, and 60% to ampicillin/sulbactam. CoNS was 100% susceptible to levofloxacin, and 75% to norfloxacin, ciprofloxacin, and ofloxacin. *S. aureus* was 100% susceptible to nitrofurantoin.

## 4 DISCUSSION

This study aimed to ascertain the prevalence of uropathogens and their corresponding antibiotic susceptibility profiles in diabetic patients receiving treatment at St. John of God Hospital, Duayaw Nkwanta, in the Bono region of Ghana. The prevalence of uropathgens among the cohort was 19.4%. This is comparable to the studies conducted in Ethiopia, Uganda, and Sudan, which reported UTI prevalent rates of 19.5%, 22.0%, and 19.5% respectively among T2DM individuals.^24^, ^25^, ^26^ Contrary to the above reports, studies conducted in the USA, Romania, Italy, and Canada found lower UTI prevalent rates of 8.2%, 12.0%, 14.9%, and 7.9% respectively.^27^, ^28^ These differences in prevalent rates may be due to differences in climatic and environmental factors that could influence the distribution of uropathogens. Also, maintaining optimal glucose levels is expensive for diabetic patients in LICs. Therefore, failure to maintain optimal glucose levels provides a suitable environment for microbial invasion, which may account for the higher prevalent rates in LICs.^24^ It is also worth mentioning that prevalent rates of different geographical areas of the same country may widely differ. For instance, while our current study found uropathogen occurrence in the Bono region of Ghana to be 19.4%, a similar study in Accra, Ghana's capital, reported a lower prevalence of 9.2%.^11^ This variation in prevalence within the same country may be due to population dynamics and resource disparities between the two areas. For instance, there are many ultra‐modern diabetic clinics and affluent people in Accra than in the remote areas like the Bono region where our study was conducted. These discrepancies have also been reported in Ethiopia and Uganda.^29^, ^30^ In terms of gender, 88.2% of the T2DM patients who tested positive for uropathogens were women. This finding is similar to the studies conducted in Ghana, Pakistan, Uganda, Iran, and Ethiopia, where rates of bacteriuria in females were respectively found to be 86.7%, 88.5%, 87.5%, 85.7, and 83.9% higher than in males.^11^, ^29^, ^30^, ^31^, ^32^, ^33^ Women's anatomical and physiological predisposition may account for this higher incidence of bacteriuria.^26^ Though studies conducted in Uganda and Germany observed a positive correlation between the age of T2DM patients and the occurrence of uropathogen,^26^, ^34^ our study did not find any significant association between age and bacteriuria. Our finding is consistent with the reports by Al‐Rubeaan et al.,^35^ Hamdan et al.,^24^ and Mama et al.^29^ Also, the occurrence of uropathogen in T2DM patients was significantly associated with neither the duration of diabetes, the type of toilet facility used nor the presence of co‐morbidities identified in this study. This observation supports the reports of similar studies conducted in Saudi Arabia and Ethiopia where the above factors did not have any association with the occurrence of uropathogens.^35^, ^36^ On the other hand, the most significant risk factor for uropathogen occurrence identified in our study was hyperglycaemia as widely reported across all the continents.^7^, ^15^, ^32^, ^37^, ^38^, ^39^ It is therefore imperative for T2DM patients to maintain optimal glucose levels to prevent the incidence of uropathogens and UTIs.

Out of the 34 culture‐positive isolates in this study, *E. coli* was the predominant (50%) followed by Gram‐positive *Enterococci* s*pp.* (14.7%) and Coagulase‐negative *Staphylococci* (11.8%). This finding corroborates several reports which identified *E. coli* as the predominant uropathogen among T2DM patients in India (53.8%), Italy (53.7%), Sudan (56.4%), Ethiopia (43.6%), Romania (68.9%), and Iran (59.1%).^24^, ^40^, ^41^ However, other studies identified *Klebsiella spp*. as the predominant uropathogen among T2DM patients in Ghana (55.6%) and Nigeria (40%) followed by *E. coli* with prevalent rates of 31.3% in Ghana and 25% in Nigeria.^16^, ^37^ These differences in the distribution of uropathogens could be due to variations in the biophysical environments in these study areas.

Furthermore, our study identified amikacin, nitrofurantoin, levofloxacin, and ceftriaxone as first‐line drugs for treating UTIs caused by gram‐negative rods. Interestingly, studies conducted in Ghana, Ethiopia, and India also found these antibiotics as the best drugs for treating UTIs caused by gram‐negative bacteria.^7^, ^15^, ^29^ It also evidenced in this study that *Klebsiella pneumonia* and *Proteus mirabilis* exhibit resistance to tetracycline, ampicillin+sulbactam, cotrimoxazole, and norfloxacin as reported from studies conducted in Ghana, Saudi Arabia, Bangladesh, Portugal, USA, Tanzania, Nepal, Pakistan, and Cameroun.^11^, ^42^, ^43^, ^44^, ^45^, ^46^, ^47^, ^48^, ^49^ Gram‐positive isolates, including *Staphylococcus aureus*, were 100% susceptible to amikacin but 100% resistant to ampicillin+sulbactam and chloramphenicol. This resistant pattern is consistent with the findings of Woldemariam et al. (2019) and Bessong et al. (2013).^7^, ^49^ The prescription of antibiotics for treating UTIs in T2DM patients receiving healthcare at SJGHDN must follow the Laboratory description of the causative organism to ensure a successful treatment outcome.

## 5 CONCLUSION

This study highlights the significant prevalence of uropathogens among diabetic patients and the concerning levels of antibiotic resistance. The findings underscore the need for careful monitoring and tailored treatment strategies for diabetic patients, especially those with risk factors such as high urine glucose levels, hazy urine appearance, and the presence of leukocytes esterase and pus cells in urine sediment. The high resistance rates to commonly used antibiotics like co‐trimoxazole and tetracycline emphasize the importance of regular susceptibility testing and the consideration of alternative antibiotics for effective treatment. These insights can guide healthcare providers in managing and preventing urinary tract infections in diabetic populations more effectively.

## STUDY LIMITATION

This study involved only 175 T2DM patients due to time and resource constraints. Since this figure represents a small fraction of T2DM patients seeking healthcare at SJGHDN per year, the study can be replicated by extending the duration and the participants so that the results can reflect the entire T2DM population of the Bono region of Ghana.

## AUTHOR CONTRIBUTIONS

Abdul‐Karim Iddrisu: Conceptualization; Methodology; Formal analysis; Software; Visualization; Writing—review and editing; Writing—original draft; Supervision; Validation; Data curation; Resources; Investigation; Project administration. George Owusu: Conceptualization; Investigation; Writing—original draft; Writing—review and editing; Visualization; Validation; Methodology; Data curation; Supervision; Resources; Software; Formal analysis; Project administration. Samuel Kofi Doe: Conceptualization; Investigation; Writing—original draft; Methodology; Visualization; Writing—review and editing; Project administration; Formal analysis; Software; Resources; Data curation; Supervision. Augustine Apraku Yeboah: Data curation; Supervision; Resources; Project administration; Formal analysis; Software; Methodology; Validation; Visualization; Writing—review and editing; Writing—original draft; Conceptualization; Investigation. Joseph Agyapong: Data curation; Supervision; Resources; Project administration; Formal analysis; Software; Methodology; Validation; Visualization; Writing—review and editing; Writing—original draft; Investigation; Conceptualization. Nicholas Yankey: Data curation; Supervision; Resources; Project administration; Formal analysis; Methodology; Validation; Visualization; Writing—review and editing; Writing—original draft; Investigation; Conceptualization; Software.

## CONFLICT OF INTEREST STATEMENT

The authors declare no conflicts of interest.

## ETHICS APPROVAL AND CONSENT TO PARTICIPATE

Ethics approval (No. CHRE/AP/157/023) for this study was granted by the University of Energy and Natural Resources Committee for Human Research and Ethics (CHRE). A well‐written informed consent form was appropriately obtained from each of the participants. The study protocol conformed to the ethical guidelines of the 1975 Declaration of Helsinki as reflected in a prior approval by the institution & # 39; human research committee.

## TRANSPARENCY STATEMENT

The lead author Abdul‐Karim Iddrisu, George Owusu affirms that this manuscript is an honest, accurate, and transparent account of the study being reported; that no important aspects of the study have been omitted; and that any discrepancies from the study as planned (and, if relevant, registered) have been explained.

## Data Availability

The data supporting the findings of this study could be made available upon reasonable request from the corresponding author.
